# Supporting Technologies for COVID-19 Prevention: Systemized Review

**DOI:** 10.2196/30344

**Published:** 2022-05-24

**Authors:** Zhuo Zhao, Rui Li, Yangmyung Ma, Iman Islam, Abdul M Azam Rajper, WenZhan Song, Hongliang Ren, Zion Tsz Ho Tse

**Affiliations:** 1 School of Electrical and Computer Engineering University of Georgia Athens, GA United States; 2 Tandon School of Engineering New York University Brooklyn, NY United States; 3 Hull York Medical School University of York Heslington York United Kingdom; 4 Department of Computer Science University of Georgia Athens, GA United States; 5 Department of Biomedical Engineering National University of Singapore Singapore Singapore; 6 Department of Electronic Engineering University of York York United Kingdom

**Keywords:** COVID-19, medical treatments, personal protective equipment, testing methods

## Abstract

**Background:**

During COVID-19, clinical and health care demands have been on the rapid rise. Major challenges that have arisen during the pandemic have included a lack of testing kits, shortages of ventilators to treat severe cases of COVID-19, and insufficient accessibility to personal protective equipment for both hospitals and the public. New technologies have been developed by scientists, researchers, and companies in response to these demands.

**Objective:**

The primary objective of this review is to compare different supporting technologies in the subjugation of the COVID-19 spread.

**Methods:**

In this paper, 150 news articles and scientific reports on COVID-19–related innovations during 2020-2021 were checked, screened, and shortlisted to yield a total of 23 articles for review. The keywords “COVID-19 technology,” “COVID-19 invention,” and “COVID-19 equipment” were used in a Google search to generate related news articles and scientific reports. The search was performed on February 1, 2021. These were then categorized into three sections, which are personal protective equipment (PPE), testing methods, and medical treatments. Each study was analyzed for its engineering characteristics and potential social impact on the COVID-19 pandemic.

**Results:**

A total of 9 articles were selected for review concerning PPE. In general, the design and fabrication of PPE were moving toward the direction of additive manufacturing and intelligent information feedback while being eco-friendly. Moreover, 8 articles were selected for reviewing testing methods within the two main categories of molecular and antigen tests. All the inventions endeavored to increase sensitivity while reducing the turnaround time. However, the inventions reported in this review paper were not sufficiently tested for their safety and efficiency. Most of the inventions are temporary solutions intended to be used only during shortages of medical resources. Finally, 6 articles were selected for the review of COVID-19 medical treatment. The major challenge identified was the uncertainty in applying novel ideas to speed up the production of ventilators.

**Conclusions:**

The technologies developed during the COVID-19 pandemic were considered for review. In order to better respond to future pandemics, national reserves of critical medical supplies should be increased to improve preparation. This pandemic has also highlighted the need for the automation and optimization of medical manufacturing.

## Introduction

The COVID-19 pandemic caused 260,221,634 confirmed cases and 5,185,350 deaths throughout the world based on data from the Coronavirus Resource Center at Johns Hopkins University, with cases continuing to rise [1]. During this unexpected pandemic, technologies have been developed in response to clinical and health care needs, pinpointed by health care workers. Examples include rapid SARS-CoV-2 test kits, low-cost ventilators, rapid sanitation methods, methods for reconfiguring hospital rooms into negative pressure isolation rooms, covers to block aerosol fluid from spreading to health care personnel during intubation and nebulization procedures, rapid-fabricated personal protection equipment and use of chest x-ray and computed tomography for COVID-19 diagnosis [2-6]. These types of solutions could rapidly address public health issues because they are easily scalable and feasible for adoption, especially in low- and middle-income countries that account for 75% of the world’s population [7]. However, there are still issues to be addressed. For example, it has been reported that the United Kingdom’s Test and Trace program is suboptimal for handling COVID-19 and its new variants [8]. Several review papers have discussed digital health technologies as a response to these issues, including artificial intelligence and big data [9-12].

In this review paper, 150 news articles and scientific reports on inventions developed to manage the COVID-19 pandemic were considered for review. From this pool of articles, technologies related to personal protection equipment, testing methods, and medical treatment were selected, resulting in a total of 23 cases for review. Each of these cases was evaluated in terms of its engineering characteristics and potential impact on the pandemic. The inventions address various problems encountered in response to COVID-19, including a lack of testing kits, the large amount of time required to obtain test results, shortages of ventilators to treat severe cases of COVID-19, insufficient accessibility to personal protective equipment (PPE) for both hospitals and the public, and the dearth of public adherence to social distancing guidelines. Some of the inventions are intended to be long-term solutions, whereas others are temporary measures. The aim of this study is to mainly focus on small to medium size supporting equipment such as facial masks and ventilators for COVID-19 prevention.

## Methods

The primary objective of this review is to compare existing supporting technologies in the suppression of the COVID-19 spread.

### Eligibility

We were interested in novel supporting technologies for COVID-19 prevention and treatment within the past 2 years.

### Exclusion

Articles were excluded if the results were published before 2020, were not in English, were not related to the event of COVID-19, were not related to mass testing and fast diagnosis, and when there was no access to the full texts.

### Searching Method

The keywords “COVID-19 technology,” “COVID-19 invention,” and “COVID-19 equipment” were used in a Google search to generate related news articles and scientific reports. The initial selection was based on the titles of the news articles and scientific reports, of which 150 articles were identified in early 2021. Another 50 articles were searched via ScienceDirect. Moreover, 5 previous review papers were included [13-15].

After the initial articles were selected, they were subjected to eliminating evaluations by 2 independently working reviewers. First, each news article, as well as scientific reports, were read and manually analyzed to remove any without a technology, invention, or equipment description, which resulted in a pool of 90 articles. Then, according to the inclusion criteria, the pool was further narrowed down to 40 articles.

Next, since some news articles or scientific reports included mentions of multiple technologies, inventions, or pieces of equipment, the initial source for each technology was tracked from the news article as well as for the scientific reports.

A final yield of 23 representative articles was obtained. These 23 articles were then divided into the three categories of personal protection equipment, testing methods, and medical treatment, which were reviewed in depth. The selection of the articles followed the guideline of PRISMA (Preferred Reporting Items for Systematic Reviews and Meta-Analyses) 2020, which can be seen in Multimedia Appendix 1 [16,17].

### Ethics Approval

Ethics approval has not been applied as there are no human participants involved in the study.

## Results

**Figure 1:**
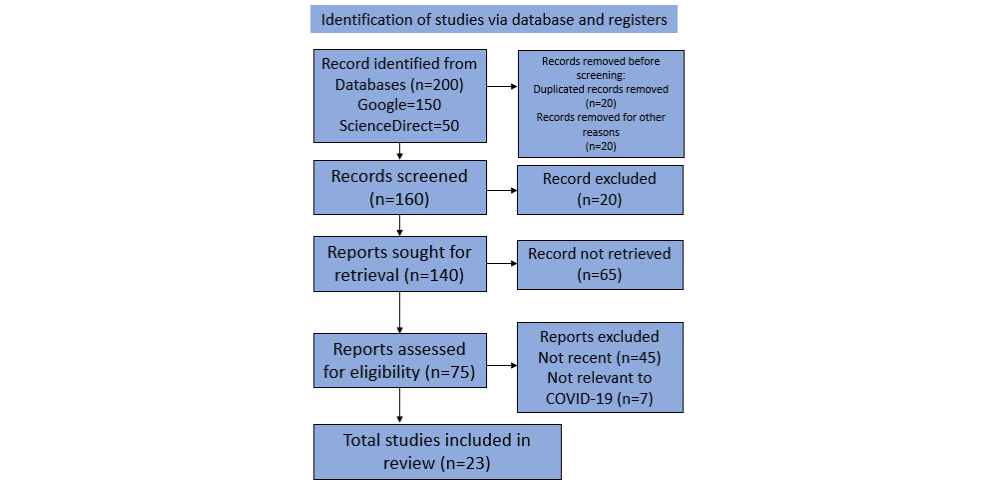
Systemized review and metanalysis flowchart.

The search results are shown in Figure 1.

### Personal Protective Equipment

Overall, 9 articles were selected for this section. In general, the design and fabrication of PPE were moving toward the direction of additive manufacturing, intelligent information feedback, and eco-friendliness

#### 3D-Printed Personal Protective Equipment

With the use of PPE becoming a necessity, the demand in the production of such equipment increased, especially when the pandemic resulted in a shortage of PPE globally [18]. This has led companies and research groups to search for faster and more efficient ways to streamline the production of, for example, face shields and masks for their worldwide distribution. To accommodate for the demand, 3D-printed face shields and masks were explored as an option due to their various advantages. 3D printing offers rapid prototyping to increase the speed and consistency of design and manufacturing, reduce cost, and maintain quality [19].

Rendeki et al [20] reviewed various PPE devices against various criteria, including 3D printing technology, materials and disinfection protocol, mechanical and structural comparison of materials used to construct PPE, and spectrophotometry. Three main models of PPE were examined, which where a half mask, a face protection shield, and safety goggles. The PPE was mainly manufactured using fused filament fabrication (FFF) technology with the purpose of filtering the air to reduce the risk of exposure to airborne diseases using an incorporated filter and fitting parameters to the face, providing an extra layer of protection to the eye. The authors reported that the PPEs that were examined were suitable as preventative measures both in safety, functionality, and durability, but disadvantages occurred mainly due to the potential hazards posed by FFF technology. This included lack of protection to the top of the head, high printing time and high material usage for the face shields, fitting problems causing leakage possibilities around the filter holder, weight problems for half masks, loss of peripheral viewing angles, and a reduced possibility of applying disinfection measures for the safety goggles. The authors mentioned these products were cost-effective only up to the break-even point of production at around 200-300 pieces. Hence, the production of additive manufacturing technologies using predominately FFF serves as a reliable but temporary solution for PPE production [20].

Amin et al [21] developed 3D-printed face shields using polylactic acid filaments, Velcro strips, adhesive foam, transparency film, and office supplies. The authors were able to print 100 face shields and distribute this locally to provide an easy and cost-effective solution for PPE; however, they noted that not all PPE devices would provide the same fluid barrier and air filtration as Food and Drug Administration–cleared PPE.

Belhouideg et al [22] and Swennen et al [19] explored several options with face masks to analyze printability and use. While the authors mentioned the ease in production, cost-effectiveness, and functionality, the importance of measuring the clinical effectiveness with regards to safety and the need for regulatory interventions were discussed.

#### Smart Personal Protective Equipment

Smart PPE offers users more information that can be used as an adjunct to further protection. It also provides information in the form of preventative measures, informing the wearer of potential risks ahead of time so that these unnecessary risks can be avoided. Other functional additions can also be included to enhance the experience of wearing such masks.

For example, Donut Robotics developed face masks that sync with a smartphone to give the user the ability to translate spoken words into text. This can be used for productivity purposes and communication and is compatible with 8 languages to accommodate a global consumer market. This may be particularly useful in a health care setting where doctors and nurses may need to communicate safely with many patients in different languages. A disadvantage may be that the translation is given in text, which may take time, and depending on the translation software used, may not be the correct translation [23].

VYZR Technologies offered a purifying shield as a response to the pandemic that provides a 360-degree seal to protect personal space on all sides. The shield has a built-in air purifying system, which is useful for filtering any pathogens, along with additional features that increase the visibility and wearability of the device. However, it was reported that the size of the shield might be inconvenient for the user and that the fan used to filter the air may be noisy [24].

Maskfone is a face mask that provides protection while allowing the user to make calls without the need to remove the mask in public. This is achieved through a built-in microphone and earphones, which reduce the inconvenience of noise pollution and ease of use. However, these masks need to be cleaned every day by changing the filter, which may be inconvenient to some users and may potentially be expensive in the long term [25].

Similarly, Airpop is a face mask that has the ability to measure breathing rate and gives alerts when it is time to change the filter. The mask is also able to track the location of the user and gives information on the quality of air and an approximate number of particles that the mask has protected the user against. These features will help track and trace those exposed to COVID-19, which has benefits of population health along with individual protection of health. However, the cost of purchasing may be significantly high and is unavailable to Android users currently [26].

Yanko Design developed a face shield with an embedded smart display that can present patients’ medical information in real time. The product also offers live recording, transfer of information, and air purifying abilities. This can be beneficial in communication, learning, and convenience between medical staff to ultimately better patient health care. However, this design is currently a concept and may require some time before it comes into production [27].

#### Environmental-Friendly Personal Protective Equipment

With the volume of disposable masks and shields produced, particularly at the beginning of the pandemic, a surge in waste disposal occurred, with the United Kingdom being responsible for a maximum of 212.5 million mask wastes per week [28]. The focus has therefore shifted toward reusable masks, which are achieved by producing masks and shields using recycled material or from household items, making them more easily washable. Such masks and shields are inexpensive and can be mass-produced, but there are concerns over safety as these masks and shields are not medically tested and may not be airtight [29,30].

Table 1 shows some major research groups or companies that are currently working on PPEs.

**Table 1:** Selected papers and major contributions.

Study groups	Countries	Descriptions	Pros and cons
Amin et al, 2021 [21]	United States	3D-printed face shields	Pros: simple; cost-effective Cons: safety concerns with the design
Swennen et al, 2020 [19]	Belgium	3D-printed face masks	Pros: ease in production; cost-effective; comfortable Cons: safety concerns with the design
Belhouideg et al, 2020 [22]	Morocco	3D-printed face masks	Pros: ease in production; cost-effective; comfortable Cons: safety concerns with the design
Donut Robotics, 2020 [23]	Japan	Speech-transcribing face masks	Pros: allowing for communication in different languages; allows spoken word to be transferred to text. Cons: prolonged translation time; potential incorrect translation due to the translation software used
VYZR Technologies, 2020 [24]	Canada	Personal air-purifying shields	Pros: 360-degree personal protection with air purifying features Cons: large; the fan may be noisy.
Maskfone, 2021 [25]	United States	Face mask with built-in earphones	Pros: allowing the users to make calls without taking the mask off; no muffled sounds Cons: a filter needs to be cleaned every day; may be costly in the long term.
Airpop, 2021 [26]	United States	Smart face mask with feedback	Pros: various features allow increased protection and prevention for the users. Cons: expensive; unavailable to Android users currently
Yanko Design, 2020 [27]	United States	Smart display face shields	Pros: increases communication, convenience, and learning opportunities through its features; real time display of information through the embedded screen
MIT Review, 2021 [30]	United States	Reusable face shields	Pros: cheap; recyclable; reusable Cons: not airtight; safety concerns

**Figure 2:**
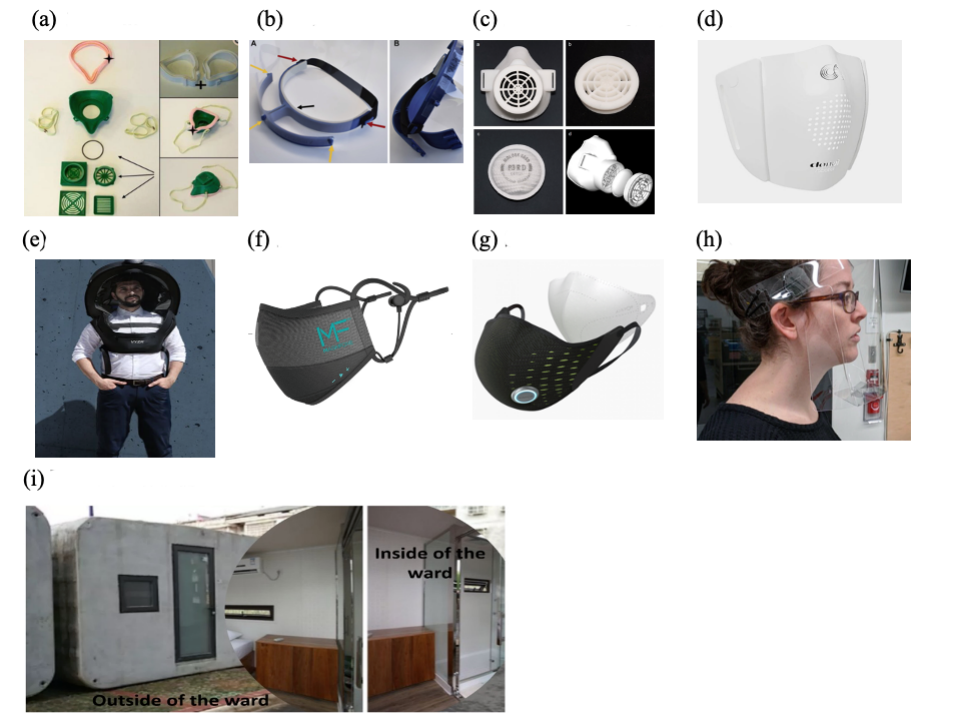
Selected equipment used in the studies: (a) 3D-printed PPE 1 [20], (b) 3D-printed PPE 2 [21], (c) 3D-printed PPE 3 [22], (d) Smart PPE 1 [23], (e) Smart PPE 2 [24], (f) Smart PPE 3 [25], (g) Smart PPE 4 [26], (h) Green PPE [30], (i) 3D-printed isolation wards from Winsun Construction Technology Co., Ltd [32]. PPE: personal protective equipment.

Figure 2 shows some selected equipment in the studies. As COVID-19 is spread through respiratory droplets, health care workers need more than protective equipment to reduce the infection risks when contacting patients. For people with mild symptoms of COVID-19, hospitalization may not be necessary. Instead, health care providers may recommend isolation at home to limit the further spread of the virus. Isolation may mean staying at home or in a designated space, remaining within a single, dedicated, adequately ventilated room, and preferably using a dedicated toilet [31]. However, this may not always be feasible since many people live with their families, where they may have to share a toilet and other communal spaces.

The Randi International think tank platform company Winsun Construction Technology Co., Ltd. has tried to overcome this problem by making 3D-printed isolation wards [32], as shown in Figure 2(i). These strong 3D wards are made from industrial and construction solid waste from urban demolition, making the wards two or three times stronger than the traditional reinforced concrete house. The wards also have an “ecological toilet” that disposes of patients’ waste without risking further spread of the virus. This solution has great scope for the future as the wards can be easily broken down, transported, and reassembled, making them ecologically protective, which will be key when outbreaks occur in new areas.

### Testing Methods

Overall, 8 articles were selected for review. The articles were divided into two main categories: molecular and antigen tests [14,33,34]. The ongoing COVID-19 pandemic outbreak has posed new challenges for public health diagnostic laboratories as the infection has become widespread internationally. Rapid and scaled-up diagnostic testing is a crucial step in slowing down the pandemic as it allows more time for treating patients before symptoms manifest and reduces the risk of patients unwittingly spreading the disease [35-37]. As such, some inventions have been developed to improve the testing speed and to optimize the testing workflow.

The traditional method of testing is for trained health care workers to collect an oral or nasal swab sample and test the sample by reverse transcription-polymerase chain reaction (RT-PCR) [8]. However, this approach currently has a major limitation as the results of the swab test are received days later, with reports suggesting that the tests are taking at least four days to return. New testing methods are required to increase the volume of tests and decrease the time taken to obtain results [38]. In Table 2, several inventions for COVID-19 testing are summarized.

**Table 2:** Selected inventions for rapid testing.

Test types and study groups		Countries	Descriptions	Pros and cons
**Molecular tests**				
	Cepheid’s Xpert Xpress SARS-CoV-2 test, 2020 [39]	United States	Automated in vitro diagnostic test for the qualitative detection of SARS-CoV-2 RNA	Pros: rapid as it is a fully automated process Cons: this test might miss several positive patient specimens.
	Abbott’s ID NOW COVID-19 rapid test procedure, 2020 [40]	United States	RT-PCR^a^ to detect nucleic acid from SARS-CoV-2 RNA	Pros: it is designed to have a small size and allow for room temperature storage. Cons: false-negative results for low positive samples
	LabCorp COVID-19 test home collection kit, 2020 [41]	United States	At-home sample collection	Pros: reduces the risk of exposure of health providers and other patients to the infection. Cons: a high false-negative result
	Accula SARS-CoV-2, 2020 [42]	United States	RT-PCR and lateral flow assay	Pros: fast turnaround, self-contained, and simple workflow Cons: the positive agreement was low for samples with low viral load.
	Cue COVID-19, 2021 [43]	United States	Isothermal nucleic acid amplification assay	Pros: very good positive and negative percent agreement with central laboratory tests Cons: about 8.6% of the initial tests need to be retested.
**Antigen tests**				
	Sofia SARS Antigen FIA^b^, 2021 [44]	United States	Immunofluorescence-based lateral flow assay	Pros: rapid results to identify patients with infection Cons: lower sensitivity
	BD Veritor System for Rapid Detection of SARS-CoV-2, 2020 [45]	United States	Chromatographic digital immunoassay	Pros: high degree of agreement for SARS-CoV-2 detection Cons: no data for the efficacy of asymptomatic population
	Abbott BinaxNOW Antigen Self-Test, 2021 [46]	United States	Immunochromatographic membrane assay	Pros: good usability Cons: test sensitivity decreased with decreasing viral loads.

^a^RT-PCR: transcription-polymerase chain reaction.

^b^FIA: fluorescent immunoassay.

**Figure 3:**
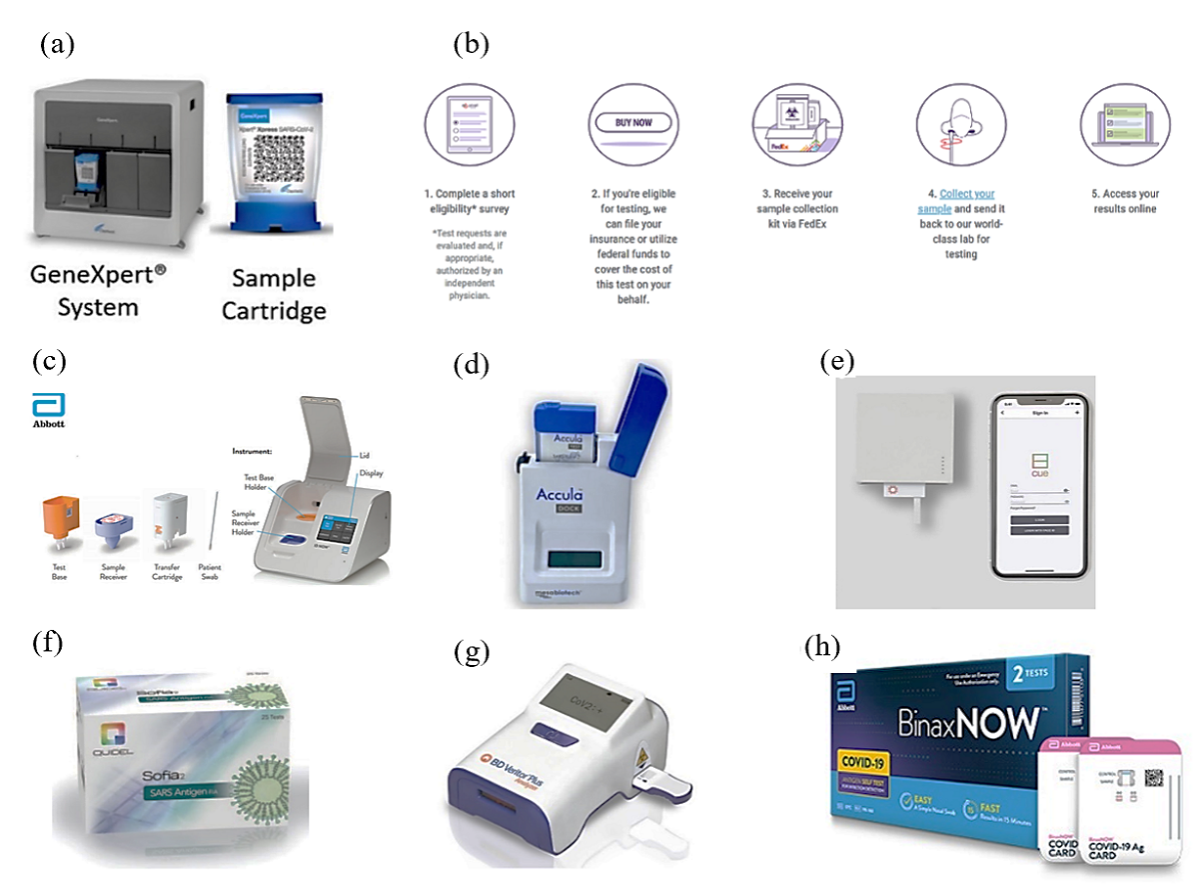
(a) Cepheid’s Xpert Xpress SARS-CoV-2 test [39], (b) the work procedure of LabCorp COVID-19 test home collection kit [41], (c) Abbott’s ID NOW COVID-19 rapid test procedure [40], (d) Accula SARS-CoV-2 test [42], (e) Cue COVID-19 [43], (f) Sofia SARS Antigen FIA [44], (g) BD Veritor System for Rapid Detection of SARS-CoV-2 [45], and (h) Abbott BinaxNOW Antigen Self-Test [46]. FIA: fluorescent immunoassay.

Cepheid’s Xpert Xpress SARS-CoV-2 test, as shown in Figure 3(a), is an automated in vitro diagnostic test for the qualitative detection of SARS-CoV-2 RNA [39]. The sample (such as a nasopharyngeal swab) is loaded into a cartridge; the cartridge is then loaded into a module; and the specimen is processed via fully automated nucleic acid extraction, amplification, amplified probe detection, and result reporting. The testing speed is rapid, enabling health care providers to obtain results within an hour of obtaining a patient sample. Once a cartridge is loaded into a module, the total time to result is about 50 minutes, with each module capable of running 28 Xpert Xpress SARS-CoV-2 tests per day. If the Xpert Xpress SAR-CoV-2 is left running, the instruments can run more than 200 patient specimens in a day [47].

Abbott’s ID NOW COVID-19 rapid test, as shown in Figure 3(c), uses RT-PCR to detect nucleic acid from SARS-CoV-2 RNA, which targets the RdRp gene [40]. It can provide positive results in 5 minutes and negative results in 13 minutes. Its small size and ability for room temperature storage enable use for testing local patients in a variety of health care environments. Patients can be tested and diagnosed on the same day as the point of care. Simple operation via visual touchscreen means it can be easily used by health care providers. Abbott is currently manufacturing 50,000 ID NOW test units per day and plans to increase its manufacturing capacity to 2 million tests per month by June 2020 [49]. However, the test is intended for testing swabs directly without elution in virus transport medium because virus transport medium samples were shown to reduce performance in low positive samples, leading to false-negative results when samples were diluted below the assay’s limit of detection [50].

In both above rapid diagnostic tests, if the virus mutates in the target region, COVID-19 may not be detected. Moreover, false-negative results may occur if a specimen is improperly collected, transported, or handled. False-negative results may also occur if there are inadequate levels of virus present in the specimen because the RT-PCR tests have a limit of detection, which is the minimum amount of viral RNA that the test will detect [51,52]. Besides improving the testing speed, optimizing the testing workflow is also helpful for increasing the testing volume and decreasing the procedure time.

Pixel by LabCorp produced a COVID-19 test, as shown in Figure 3(b), that allows for at-home sample collection. Patients can self-swab to collect their nasal samples and mail their samples in an insulated package to a LabCorp lab for testing. It allows for sample collection within the safety of the home and is beneficial because it reduces the risk of exposure to health providers and other patients to the infection [41]. It would also cut down on demand for PPE that is needed to collect specimens using the traditional testing method [53]. Test kits can be deployed on a large scale so masses of the population can be tested to help slow the spread of COVID-19. However, this self-collection kit could cause a high false-negative result if some customers perform the collection procedure incorrectly [54,55].

In the category of antigen test, the Sofia SARS Antigen FIA (fluorescent immunoassay) uses sandwich immuofluoresence-base lateral flow for the qualitative detection of SARS-CoV-2 nucleocapsid protein antigen. Based on the clinical evaluation, there was a significant reduction in turnaround time from sample collection to test results. Compared to RT-PCR, the turnaround time was reduced from 20.1 hours to 1.2 hours for Sofia SARS Antigen FIA. However, a previous study also suggested that antigen test is less suitable for both very early and late stages of SARS infection as it has lower sensitivity at high cycle threshold values [44]. The other 2 antigen tests are BD Veritor System and Abbott BinaxNOW antigen self-test [48,56].

### Medical Treatments

Overall, 6 articles were selected for review. The major challenge was how to apply novel ideas to speed up the production of ventilators. Ventilators are a form of life support that takes over the work of breathing when a person is not able to breathe enough air on their own [57]. Individuals who develop COVID-19 are at risk of developing serious lung complications such as pneumonia and, in severe cases, acute respiratory distress syndrome [58,59]. In severe cases, which account for 1 in 6 people, patients require ventilatory assistance. Governments have become increasingly aware of the demand for ventilators and have started upping production [60]. For example, the United Kingdom has added another 8000 ventilators to their existing 8000, while the United States estimates it will need 60,000-160,000 additional ventilators [61,62]. Table 3 shows some major research groups or companies that are currently working on ventilators.

**Table 3:** Selected papers and major contributions.

Study groups	Countries	Descriptions	Pros and cons
MIT, 2020 [63]	United States	“Bridge” ventilators that automate manual resuscitators	Pros: aid breathing for less acute patients Cons: N/A^a^
Virgin Orbits, 2020 [64]	United States	“Bridge” ventilators that automate manual resuscitators	Pros: aid breathing for less acute patients Cons: N/A
Glangwili Hospital, 2020 [65]	United Kingdom	Snood-type mask	Pros: rapid production Cons: N/A
Materialize, 2020 [66]	Belgium	Positive end-expiratory pressure for patients without a true ventilator.	Pros: rapid assembly as it is 3D printed Cons: N/A
OxVent ventilator, 2020 [67]	United Kingdom	Built from off-the-shelf components	Pros: portable and scalable Cons: not under rigorous quality test
Patients-shared ventilator, 2020 [68]	United States	Accommodate 2 patients at the same time	Pros: maximize the usage of valuable hospital equipment Cons: potential health and safety risk

^a^N/A: not applicable.

**Figure 4:**
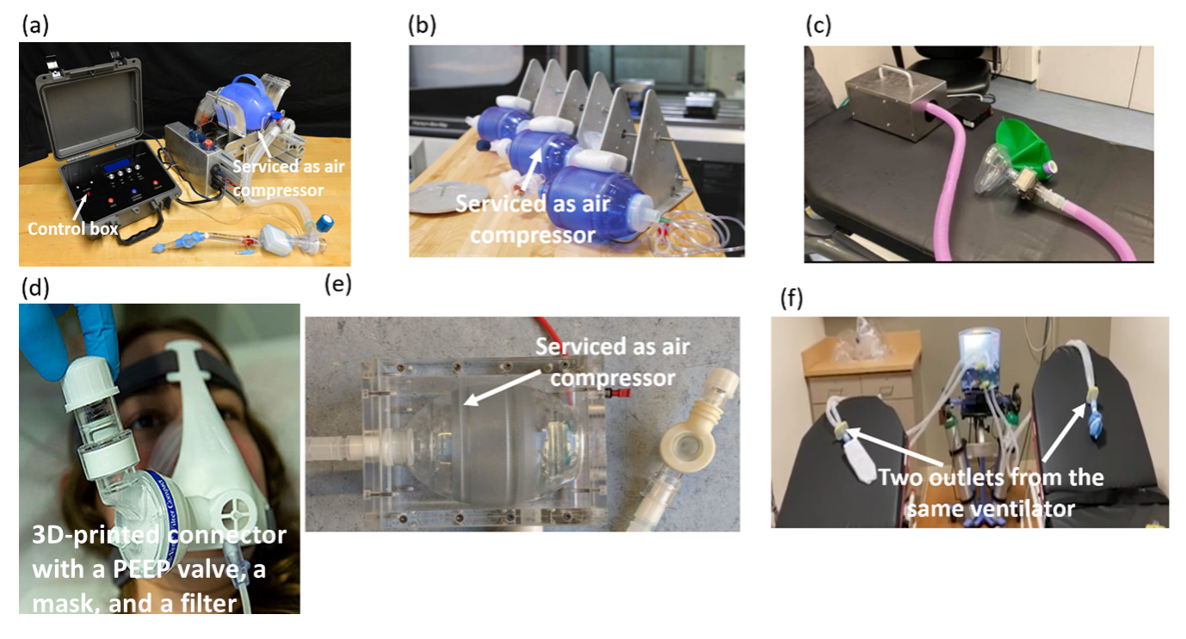
Ventilators designed by (a) MIT [63], (b) Virgin Orbits [64], (c) Glangwili Hospital [65], and (d) Materialize [66]. (e) OxVent ventilator [67]. (f) Patient-shared ventilator [68]. PEEP: positive end-expiratory pressure.

With the heavy demand for ventilators, researchers and companies have started to design highly scalable, innovative ideas to match these demands. MIT and Virgin Orbits have designed similar “bridge” ventilators that automate manual resuscitators, as shown in Figure 4(a) and 4(b); they aim to aid breathing for less acute patients, therefore alleviating the use of current ventilators in intensive care units [63,64]. Furthermore, a group from Glangwili Hospital is using mechanical technology to build a snood-type mask, as shown in Figure 4(c), which is connected to a filter to purify the air of coronavirus particles and to supply it to the user [65].

Materialize developed a technology to provide positive end-expiratory pressure for patients without a true ventilator. A source of oxygen was the only requirement to achieve ventilator function [66]. As shown in Figure 4(d), the design consists of a 3D-printed connector that connects a positive end-expiratory pressure valve, a mask, and a filter. A multidisciplinary team of engineers and medics at the University of Oxford and King’s College London have developed a new ventilator called OxVent, as shown in Figure 4(c), which is made from off-the-shelf components and equipment with certain elements that can be produced through 3D-printing techniques [67]. The OxVent is portable and inflates the patients’ chest by injecting compressed air. Another solution to respond to the shortage of ventilators was developed in several hospitals, where they shared the same ventilator between 2 patients with some normal tubes instead of building a new ventilator, as seen in Figure 4(f) [68]. An operation protocol for ventilator sharing has been developed by engineers and medics as a response to this innovation to ensure safety [69].

## Discussion

### Principal Findings

The shortage of medical equipment, such as masks and ventilators, has been the biggest challenge. Although insufficient stockpiling of medical equipment is one of the reasons attributed to the shortage, the most important reason is the high labor dependency of the medical equipment industry. The shortage of labor and the high infection risk in a crowded working environment have limited the capacity of the medical equipment industry. Approaches should be considered for the medical equipment industry for future responses. It is time to optimize the current processing flow and improve the degree of automation so that the dependence on labor can be reduced. Many cases have been reported on the use of 3D printers for producing medical equipment on a small scale, such as masks, ventilator parts, and quarantine rooms. Although 3D-printing technology could significantly reduce the amount of labor required for production, cost and efficiency are still challenges at this stage. Other critical labor-intensive industries, such as the food processing industry and delivery industry, have also been reported as imposing high risks of large-scale infection [70-72]. Labor shortages in these industries have led to shortages of basic human necessities. For such industries, improving the degree of automation and reducing the degree of labor dependence are also necessary measures to ensure better responses to future pandemics.

On the other hand, the shortage of lifesaving machines such as ventilators during the pandemic could illustrate a point: medical technology research, supported by taxpayer money, may not be sufficient for handling global outbreaks such as COVID-19. Traditional funding mechanisms have singularly focused on supporting “high-risk, high-reward” research activities to support creative scientists pursuing highly innovative research rather than low-cost and scalable technologies that could address the public health demands during the pandemic. Technologies developed to address the COVID-19 pandemic should meet epidemiological needs and help manage outbreaks. They need to be low-cost scalable solutions that are practical for patients and health care workers as well as being widely accessible to the global community. However, publicly funded medical research has long been skewed toward ideas proposed by research-intensive, highly developed, and resource-abundant researchers.

### Comparison With Prior Studies

#### Personal Protective Equipment

PPE is an intervention that has become a necessity as a first-line preventative method against the pandemic, and the culture of wearing PPE, particularly the wearing of face masks, may continue in the long run [28]. In the health care setting, the wearing of PPE may become an indefinite feature, and therefore, the development of PPE, particularly in terms of safety and convenience, may be of paramount importance. Current developments have focused on streamlining the production of PPE in preparation for future pandemics, increasing the convenience and experience of the wearer, and making the production of the PPE more sustainable by using reusable resources [18,29]. While these are exciting prospects, researchers and developers must not forget that developing the protection provided by PPE is the most important feature. The developments mentioned in this article still require approval from governing bodies with regards to safety and, therefore, must continue to focus on producing PPE that is in line with the guidelines set by governing bodies with regards to acceptable requirements of protection [18,73]. The other challenge is producing the aforementioned PPE developments on a large scale and at a low cost. While currently this may be difficult, technological considerations toward reducing production costs to increase the accessibility of products may be beneficial.

#### Testing Methods

One challenge among the inventions developed to slow the spread of COVID-19 is the ability to pass rigorous scientific testing. The inventions reported in this review paper have not been sufficiently tested for their safety and efficiency. Most of them are temporary solutions intended to be used only during shortages of medical resources. However, these medical devices still need Food and Drug Administration approval before they can be offered as commercial products on the market. Many prospective COVID-19 inventions will likely be rejected for safety reasons. Lessons can be learned from this pandemic to serve as guidance to improve the response to future pandemics and outbreaks.

#### Medical Treatment

The implementation of highly technological solutions, which require long-term development and expensive setup in pandemic response, may face many obstacles. While robotic technologies have great potential as tools to meet specific clinical needs, robots are unlikely to be widely adopted for COVID-19–related applications due to cost and manufacturing time. Robots capable of unique tasks need to meet epidemiological requirements, which could be costly, impractical, and most likely accessible only to the wealthiest hospitals and businesses, which means only a small proportion of people can receive the benefit. Investigating solutions to the pandemic shall consider underprivileged communities that are most vulnerable to both infection and continued transmission. Furthermore, tools for outbreak control need to be mass-produced and distributed quickly; however, with the exponential spread of SARS-CoV-2 around the globe, the time required to fabricate complex robotics would be prohibitive to this acute demand.

One possible area to improve is the ability to provide appropriate palliative care. Radbruch et al [74] discuss the importance of palliative care in the COVID-19 response. They highlight the need for two key measures to be taken throughout the world: first, to increase national reserves of opioid medications while controlling costs by implementing pooled purchasing platforms, and second, to provide basic palliative care training to all primary caregivers and health care professionals in emergency departments and intensive care units [74]. This type of response is practical because it addresses the need for public health responses to COVID-19 to be inexpensive and widely accessible.

#### Other Considerations

One challenge in this pandemic is the high infection rate of health care workers [73,75], which has led to a shortage of health care workers [76,77]. The high infection rate is caused by the close contact between health care workers and patients during diagnosis and treatment, so it is important to reduce contact in order to reduce the infection rate of health care workers in future pandemics. Two methods could be used to achieve this purpose. The first is to optimize the current diagnosis workflow and environment. Remote prediagnosis through the internet or phone could increase the work efficiency of health care workers and reduce contact time. Separate pathways and rooms for patients and doctors could be set up in areas of high transmission risk to reduce the amount of shared area and thus eliminate unnecessary contact. The second is to apply more medical robotics in the treatment process. Medical robotics could enable social distancing between patients and doctors during treatment. In addition, robotics could help improve the efficiency of health care workers; for example, tracheal intubation currently requires 3 people, but it could be done by 1 person with assistance from medical robotics [4]. Besides medical robotics, other types of robotics can be applied in hospitals for sterilization, drug or food delivery, sample transfer, and diagnostic testing [78-81].

### Study Limitations

The paper only investigated the small to medium size supporting medical equipment for COVID-19. Large equipment such as computed tomography or magnetic resonance imaging scanners have not been included in this study. Moreover, the paper only provides a qualitative comparison between the technologies. The search strategy was not comprehensive as it was limited to two databases: Google and ScienceDirect. Even though some of the complexities were unveiled regarding supporting technologies, a quantitative analysis would have also added value to the review results. Moreover, the protocol that was not registered with PROSPERO (international prospective register of systematic reviews) might have affected the results in one way or the other. There was no formal appraisal of the included studies as well as the overall evidence from included studies.

### Conclusion

The study objectives were to evaluate existing support technologies for COVID-19 prevention, diagnosis, and treatment. A total of 18 technologies in the areas of PPE, testing methods, and medical treatment were selected for review. The engineering characteristics of each invention were summarized, and the potential to make a significant impact on the pandemic response was evaluated and discussed. One major hurdle to adopting the technologies discussed in this paper or any other prospective technologies was that COVID-19–related research is still in the early stages, so even if innovations look promising, their safety and efficiency have not yet been tested and evaluated in a rigorous scientific manner.

The unexpectedly large and widespread impact of the COVID-19 pandemic has led to many challenges in the management of the disease for both public health agencies and hospitals. Shortages of essential medical resources, including SARS-CoV-2 testing kits, ventilators, and personal protective equipment, have been the biggest challenge throughout the world. In this review paper, technologies developed during the COVID-19 pandemic in response to clinical and public health needs were considered for review. In order to better respond to pandemics in the future, several directions have been discussed. For example, national reserves of critical medical supplies should be increased to improve preparation. Regarding the manufacturing of medical equipment, this pandemic has highlighted the need for the automation degree of medical manufacturing to be increased and for production workflows to be optimized. Finally, a shift in the approach to funding scientific research should be implemented during pandemics to promote low-cost, scalable solutions.

## Appendix

Multimedia Appendix 1 PRISMA (Preferred Reporting Items for Systematic Reviews and Meta-Analyses) 2020.

## Abbreviations

FFF: fused filament fabrication
FIA: fluorescent immunoassay
PPE: personal protective equipment
PRISMA: Preferred Reporting Items for Systematic Reviews and Meta-Analyses
PROSPERO: International Prospective Register of Systematic Reviews
RT-PCR: reverse transcription-polymerase chain reaction
